# Giant myxoid liposarcoma of the stomach: Report of a case

**DOI:** 10.1016/j.ijscr.2019.06.025

**Published:** 2019-06-20

**Authors:** Akari Sonoda, Hiroshi Sawayama, Nobutomo Miyanari, Takao Mizumoto, Tatsuo Kubota, Hideo Baba

**Affiliations:** a Department of Surgery, National Hospital Organization Kumamoto Medical Center, Japan; b Department of Gastroenterological Surgery, Kumamoto University Graduate School of Medical Sciences, Japan

**Keywords:** Myxoid liposarcoma, Stomach, Surgery, Case report

## Abstract

**Introduction:**

Liposarcoma is one of the most common soft tissue sarcomas in adults, but liposarcoma arising primarily from the stomach is very rare.

**Presentation of case:**

A 56-year-old man was referred to our hospital with abdominal distension and discomfort. A computed tomography scan showed a huge mass located between the stomach and transverse colon. The preliminary differential diagnoses were sarcoma with a mucinous component, gastrointestinal stromal tumor, lymphangioma, and mesenteric cyst. Upper and lower endoscopy was not performed because the patient declined. The patient underwent surgical resection, and the tumor was completely removed. Macroscopic examination revealed a 39- × 26- × 20-cm tumor weighing 13,000 g. On histological examination, the tumor was diagnosed as a myxoid liposarcoma in the gastric submucosa. The patient was still doing well 2 years postoperatively.

**Discussion:**

Preoperative diagnosis of liposarcoma of the stomach is difficult. In the present case, the tumor was very large but was able to be resected completely.

**Conclusion:**

Even in patients with very large myxoid liposarcoma, curative resection can provide a good prognosis.

## Highlights

• Myxoid liposarcoma of the stomach is extremely rare.
• The tumor in the present case was too large to confirm its origin.
• Imaging findings of liposarcoma vary, and few reports have described gastric liposarcoma with a huge cyst.
• Even for large tumors, curative resection can provide the patient a good prognosis.

## 1 Introduction

Liposarcoma is one of the most common soft tissue sarcomas in adults. The latest research in the United States showed that liposarcoma was the second most frequent type of sarcoma after undifferentiated pleomorphic sarcoma, and leiomyosarcoma ranked third [1]. Leiomyosarcomas are the most common type of sarcoma found in the abdomen, while liposarcomas are the most common type in the lower extremities (56%) and retroperitoneal region (20%) [2]. Gastric myxoid liposarcoma is very rare, and only a few cases have been reported worldwide [3]. Because this tumor usually grows slowly with expansive rather than infiltrative behavior, symptoms usually emerge late and remain unnoticed until the tumor is quite large. Tumor resection is the only curative treatment for liposarcoma. If the tumor is too large, its boundary might not be identified by radiological examination. This often makes surgical management difficult. This case report provides information on the clinical course, imaging outcomes, pathological features, and treatment of a giant myxoid liposarcoma of the stomach. This work has been reported in line with the SCARE criteria [4].

## 2 Case presentation

**Fig. 1:**
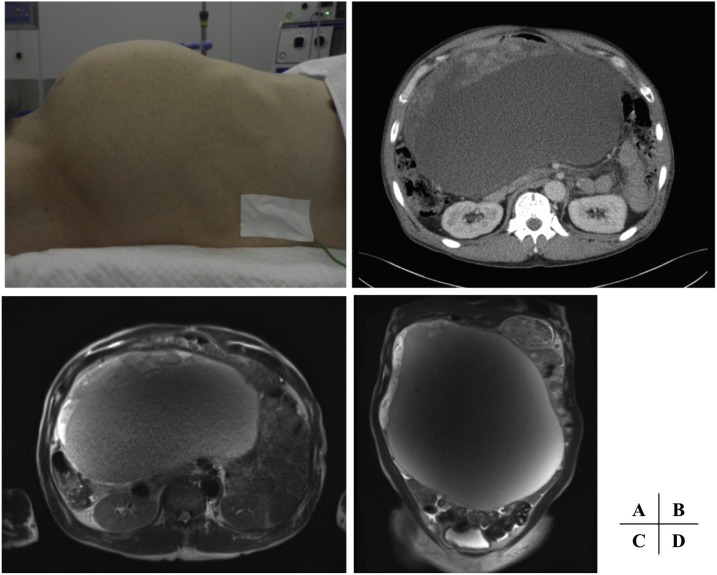
A. Distended abdomen before surgery. B. Computed tomography revealed the large cyst and solid component showing enhancement. C, D. T2-weighted magnetic resonance imaging also showed the large cyst and solid component with high signal intensity.

A 56-year-old man was referred to our hospital with a 6-month history of abdominal distension and discomfort (Fig. 1A). He had a medical history of schizophrenia. His abdomen was markedly distended, and severe edema was present in both lower limbs. He had no symptoms of gastrointestinal obstruction. Neurological examination findings were normal. He had slight anemia (hemoglobin of 10.5 g/dl), and other laboratory data were within normal limits. A computed tomography (CT) scan demonstrated a 30- × 18- × 30-cm giant mass located between the stomach and transverse colon. It included a large cyst and solid component that showed enhancement (Fig. 1B). The main feeder artery for the tumor seemed to be the right gastric artery. Magnetic resonance imaging (MRI) also showed a huge heterogeneous soft tissue mass. The solid component showed high signal intensity on T2-weighted imaging and diffusion-weighted imaging (Fig. 1C, D). Upper and lower endoscopy was not performed because the patient declined. CT-guided biopsy was not performed to avoid dissemination. Our preoperative differential diagnoses were sarcoma with a mucinous component, gastrointestinal stromal tumor, lymphangioma, and mesenteric cyst. A histological diagnosis was not obtained preoperatively, and the tumor was too large to identify its boundary with the surrounding organs by radiological examination. We expected that the tumor was arising from the stomach, transverse colon, or mesenterium.

**Fig. 2:**
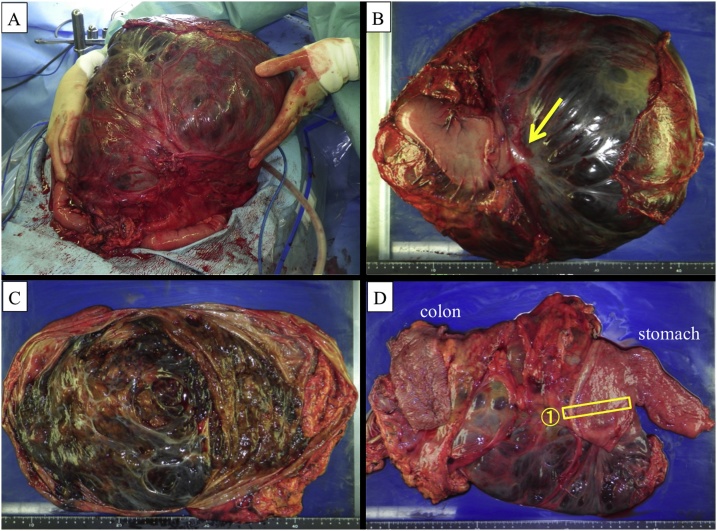
A. We gradually dissected along the capsule of the caudal side of the tumor, and most of the tumor was able to be moved outside the body. B. The resected specimen weighed 13,000 g and measured 38 × 20 × 19 cm. Part of the stomach wall seemed to be pulled by the tumor (arrow). C. The cyst was opened and examined from the inside. D. No abnormalities were found on the intraluminal surface of the stomach and transverse colon wall. ① Indicates the arrow portion of picture B.

We decided to perform surgery because the tumor showed the tendency to grow. During laparotomy, we identified a huge encapsulated tumor. The tumor occupied most of the pelvic cavity, but the caudal side of the tumor had no adhesions with pelvic organs. We gradually dissected along the capsule and moved the tumor outside the body (Fig. 2A). Finally, we found that the tumor was adhered to the stomach and transverse colon. We resected the distal stomach and 15 cm of the transverse colon with the tumor. Reconstruction was performed using Billroth-I anastomosis for the stomach and end-to-end anastomosis for the colon. We identified the resection margin of the tumor, and we did not perform intraoperative histological examination or lymph node dissection because no enlarged lymph nodes or disseminated nodules were found. The tumor was completely removed. The postoperative course was uneventful, and the patient was discharged on postoperative day 17.

**Fig. 3:**
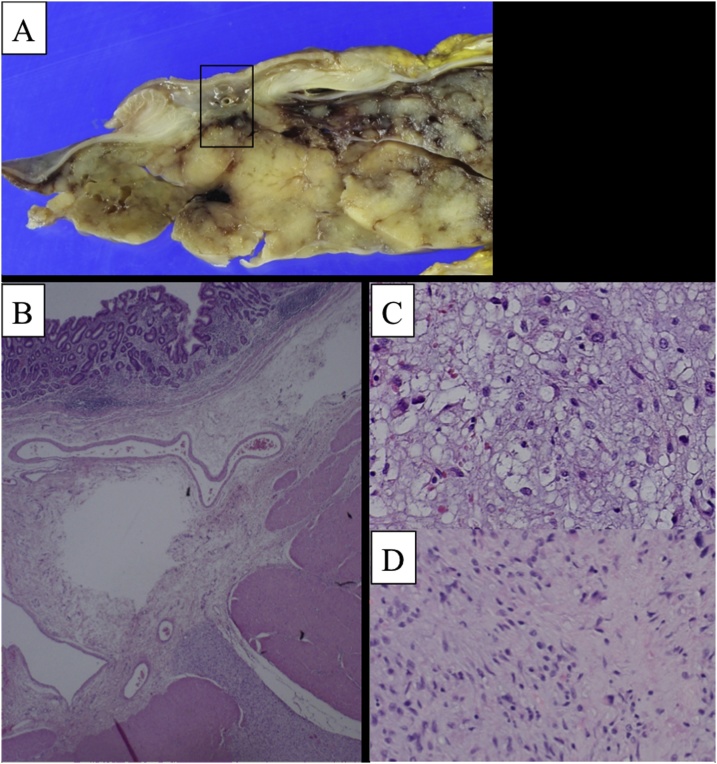
A. Cut surface of ①. The solid component of the tumor showed continuity with the stomach wall. B. Tumor cells developed from the submucosa of the stomach and grew extraluminally. C, D. The tumor was composed mainly of short spindle and vacuolated cells, including lipoblasts and mature adipocytes, with a myxomatous matrix.

Macroscopic examination revealed a 38- × 20- × 19-cm tumor weighing 13,000 g (Fig. 2B). No abnormalities were found on the intraluminal surface of the stomach or transverse colon wall. On histological examination, the tumor was composed mainly of short spindle and vacuolated cells, including lipoblasts and mature adipocytes, with a myxomatous matrix. The main mass was located in the abdominal cavity, but the tumor base was broadly adhered to the gastric wall and seemingly grew from the gastric submucosa, suggesting that the tumor had likely arisen from the stomach (Fig. 3). The transverse colon was intact. Immunohistochemically, the tumor cells were negative for smooth muscle actin, c-kit, and MDM2. These features were consistent with myxoid liposarcoma. The patient was still doing well 2 years postoperatively.

## 3 Discussion

We treated a rare case of myxoid liposarcoma arising from the stomach. The tumor was very large but was able to be resected completely. Curative resection may provide a good prognosis for patients with myxoid liposarcoma.

Liposarcoma is one of the most common soft tissue sarcomas in adults, constituting 20% of all soft tissue sarcomas. It usually occurs in the soft tissues of the extremities and retroperitoneum [2,5]. Intra-abdominal liposarcomas that grow slowly and cannot be seen from the body surface are often detected in the late stages. Liposarcoma is histologically defined as a tumor composed of lipoblasts. According to the 4th edition of the World Health Organization Classification of Tumours–Pathology and Genetics of Tumours of Soft Tissue and Bone, the four main morphologic subgroups are well-differentiated/atypical lipomatous tumor (50% of all liposarcomas), myxoid liposarcoma (30%), pleomorphic liposarcoma (5%–10%), and dedifferentiated liposarcoma. The well-differentiated type has a low risk of metastasis, and the myxoid and pleomorphic types have a higher risk of metastasis. The myxoid subtype, which includes round cells, has a particularly higher risk of metastasis and recurrence after surgery [6].

Preoperative diagnosis of liposarcoma is often difficult. In our case, CT demonstrated a large cyst and solid component that showed enhancement, and MRI demonstrated a huge heterogeneous soft tissue mass. The solid component showed high signal intensity on T2-weighted MRI. The features of a heterogeneous mass with minimal fat are suggestive of liposarcoma, and myxoid liposarcoma generally has the features of feathery enhancement and a high fluid content [7]. Liposarcoma of the stomach in which the cystic component occupies the majority of the tumor, as in our case, is rare, and huge cystic liposarcomas similar to our case but arising in the retroperitoneum or descending colon have also been reported [8,9]. We did not perform endoscopy at the patient’s request, and we could not confirm the histological diagnosis preoperatively. About 40 cases of liposarcoma of the stomach have been reported to date, and two preoperative histological diagnoses were confirmed by endoscopic biopsy. Most biopsy specimens were diagnosed as inflammatory mucosa, erosion, or lipoma [3,[10], [11], [12], [13]].

Surgery is the basic treatment for liposarcoma [14]. An appropriate surgical strategy is very important for safe and complete resection of giant tumors. *En bloc* and complete resection contribute to a good prognosis [15]. A previous report described a case in which a 14-cm retroperitoneal tumor was laparoscopically resected [16], but we chose laparotomy because our patient had a tumor diameter of ≥30 cm. Our case involved a large tumor that occupied the pelvic cavity, and we found that the tumor was adhered to the distal stomach and transverse colon during surgery. We predicted that one of the organs was the tumor origin and therefore performed *en bloc* resection to reduce the risk of local recurrence [17]. It is often difficult to determine the origin of the tumor by preoperative radiological examination; thus, the surgical procedure needs to be chosen intraoperatively. Radiation and chemotherapy are other treatments for liposarcoma. Radiation therapy is reportedly effective for myxoid liposarcoma in the extremities [18]. When a sufficient resection margin cannot be secured, postoperative irradiation may be performed to prevent recurrence [19]. We did not perform adjuvant therapy in our case because no residual tumor was present.

## 4 Conclusion

We have herein described a rare case of treatment of a giant myxoid liposarcoma arising from the stomach. Surgery is the only therapeutic option in such cases. If the tumor can be resected completely, even a very large tumor, the patient can achieve long-term survival.

## Conflicts of interest

The authors declare that they have no conflicts of interest.

## Sources of funding

This study did not receive any funding support.

## Ethical approval

This is a case report; therefore it did not require ethical approval from ethics committee.

## Consent

Written informed consent was obtained from the patient.

## Author’s contribution

Akari Sonoda contributed acquisition of clinical data, drafting of the manuscript.

Hiroshi Sawayama contributed acquisition of data, drafting of the manuscript.

Nobutomo Miyanari contributed surgical procedures of this case report.

Takao Mizumoto contributed critical revision of the manuscript.

Tatsuo Kubota contributed critical revision of the manuscript.

Hideo Baba contributed study supervision.

## Registration of research studies

Researchregistry4735.

## Guarantor

The Guarantor of this study is Hideo Baba.

## Provenance and peer review

Not commissioned, externally peer-reviewed.
